# Mechanism, research progress and warning of the effects of acupuncture at Zusanli point (ST36) on pharmacokinetics: A review

**DOI:** 10.1097/MD.0000000000045635

**Published:** 2025-10-31

**Authors:** Xinyu Liu, Wenfeng Zhang, Yifan Zhao, Cheng Li, Yonggang Wang, Yinghua Hu

**Affiliations:** a School of Basic Medical Sciences, Changchun University of Chinese Medicine, Changchun, Jilin, China; b Department of Cardiovascular Center, The First Hospital of Jilin University, Changchun, Jilin, China; c College of Acupuncture and Tuina, Changchun University of Chinese Medicine, Changchun, Jilin, China.

**Keywords:** acupuncture, acupuncture-drug interaction, pharmacokinetics, ST36, systematic review

## Abstract

In traditional Chinese medicine, acupuncture is considered free of harmful side effects when properly performed. Pharmacokinetics provides a valuable approach to explore how acupuncture influences drug disposition. Stimulation at specific acupoints, such as Zusanli (ST36), may modulate drug absorption and action. While most studies highlight beneficial outcomes, potential risks also deserve consideration. A systematic review was conducted to evaluate how acupuncture or electroacupuncture at ST36 affects the pharmacokinetics of various drugs. Data on changes in absorption, distribution, metabolism, and excretion, along with pharmacokinetic parameters (e.g., average plasma concentration-time curve [AUC], *C*_max_, *T*_max_), were extracted and analyzed. Acupuncture at ST36 significantly influenced the pharmacokinetics of several compounds. For instance, it enhanced the absorption and plasma levels of Schisandra chinensis lignans and acetaminophen (APAP), increasing AUC and *C*_max_ while shortening *T*_max_. Tissue distribution also shifted, with elevated concentrations observed in certain organs such as the lungs. While aspirin metabolism remained unaffected, ST36 stimulation delayed APAP clearance at higher doses, resulting in prolonged systemic exposure. In the case of triptolide, a bioactive diterpenoid, acupuncture increased its plasma levels and AUC. Some studies reported changes in biomarkers associated with liver function under elevated compound exposure, though no acute adverse effects were noted for commonly used agents. ST36 acupuncture can alter drug pharmacokinetics, potentially impacting drug exposure and clinical response. Awareness of such interactions is important when combining acupuncture with medication. Further studies are warranted to ensure the safe and effective integration of acupuncture into pharmacological treatment strategies.

## 1. Introduction

Traditional Chinese medical theory posits that the human body has inherent channels called meridians, which connect organs and tissues. Qi and blood, the fundamental substances sustaining life, circulate within these meridians. Specific points along the meridians (acupoints) have unique effects by linking to tissues and organs. Under physiological conditions, this system maintains balance; however, pathological changes can alter the meridian–acupoint–organ network. For example, heart disease may manifest along associated meridians and acupoints with symptoms such as pain, redness, or numbness. Early healers found that stimulating these affected areas could relieve symptoms and treat diseases, leading to the development of acupuncture. Acupuncture, emerging in the Neolithic period and evolving over centuries, typically uses filiform needles inserted into acupoints to modulate Qi and blood flow and thereby influence organ function.^[1–4]^ Modern research has elucidated that acupoint stimulation exerts effects through neuroendocrine-immune feedback pathways.^[5–8]^ For instance, stimulation of Zusanli (ST36) transmits signals to the brain via peripheral nerves; the brain in turn sends regulatory signals that can affect immune cells, neurotransmitters, and autonomic nerves. One observed effect is enhanced gastrointestinal motility via vagus nerve activation, which can alter drug absorption and metabolism. These findings illustrate 1 aspect of a highly intricate regulatory system.^[5,7,9–12]^ Neuroimaging studies further show that acupuncture can modulate specific functional areas of the brain. Differences in brain activation patterns between true acupoint stimulation and non-acupoint stimulation suggest specific connections between acupoints and neural circuits. Factors such as sex and physiological state also influence responses to acupuncture.^[13–15]^ When patients receive acupuncture alongside medications, the combined effects on the body may be unpredictable. While acupuncture is generally perceived as beneficial and safe, its potential to modify drug effects has been underexplored. Most safety studies focus on acupuncture’s direct side effects and do not address risks when acupuncture and medications are used together.^[16–20]^ In practice, many patients receive acupuncture concomitantly with herbal or Western medicines. It is plausible that acupoint stimulation could affect drug pharmacokinetics, potentially causing changes in drug efficacy or adverse reactions. Therefore, it is crucial to objectively evaluate how acupuncture might interact with drugs. This review aims to summarize known effects and mechanisms of acupuncture at ST36 on drug pharmacokinetics, to raise awareness of possible acupuncture–drug interactions without unwarranted alarm.

Pharmacokinetics provides a concrete framework to study how acupuncture influences drugs in the body. There is extensive research on drug–drug and herb–drug interactions, but studies on acupuncture–drug interactions remain limited. We collected and analyzed studies on acupuncture or electroacupuncture at ST36 affecting drug absorption, distribution, metabolism, and excretion. Our goal is to elucidate the mechanisms of these effects and improve understanding of the potential impact of acupuncture on drug therapy, while maintaining an objective perspective. We particularly aim to identify any significant changes in drug exposure caused by ST36 stimulation and to consider their clinical implications.

## 2. ST36

**Figure 1.**
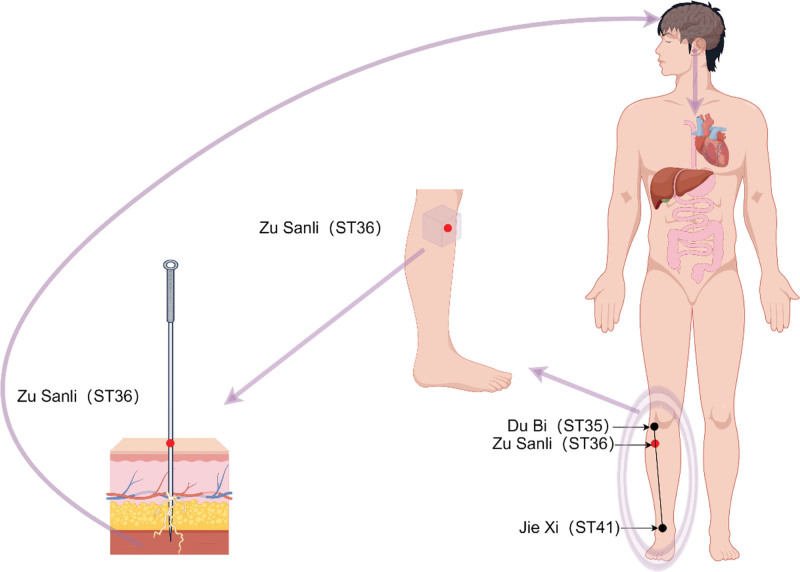
Schematic diagram of the surface position and the deep anatomical structure of ST36 during acupuncture insertion. The reaction of the human body after the needle is inserted into Zusanli.^[33–38]^

In traditional medicine, ST36 is located on the stomach meridian of Foot Yangming. It is situated on the anterior aspect of the calf, along the line connecting Dubi (ST35) and Jiexi (ST41), approximately 3 inches below ST35. This region has a rich distribution of nerves and blood vessels.^[21]^ Studies have shown that stimulating ST36 has an impact on the digestive, immune, nervous, and respiratory systems, as well as energy metabolism and psychological diseases.^[22–32]^ It is one of the most widely used acupoints in clinical practice^[33–38]^ (Fig. 1).

Therefore, the mechanism of ST36 in treating diseases has been extensively studied. An old Chinese saying likens ST36 to “an old hen that costs nothing (old hens have a nourishing effect in the minds of the Chinese people),” reflecting the Chinese people’s understanding of the efficacy of ST36. Despite its familiarity, the potential risks associated with its use are often overlooked. ST36 has a wide range of therapeutic effects and is commonly used in clinical practice. Previous studies^[26,39–47]^ have shown that ST36 can treat ischemia-reperfusion injury, heart dysfunction, diabetes and its complications, digestive dysfunction, nervous system damage, and psychological diseases.

In clinical practice, it is common to use acupuncture and medication simultaneously on the same individual.^[48–61]^ Therefore, the risk of acupuncture ST36 has also increased, which has attracted our attention and research. While this concern extends to all acupoints, the widespread use and prominence of ST36 make it an ideal focus for raising awareness about these risks.

## 3. Effect of acupuncture on pharmacokinetics

Pharmacokinetics is a fundamental concept in pharmacology and an important indicator for understanding the effects of drugs on the human body. It quantitatively studies the processes drugs undergo in the body (absorption, distribution, metabolism, and excretion) using mathematical principles to explain the dynamic behavior of drugs. Determining the appropriate dosage and interval of a drug relies on whether it can achieve a safe and effective concentration at its site of action. Drug metabolism can be assessed by measuring the concentration of the drug or its metabolite in body fluids such as serum and urine.^[62]^ These processes are influenced by various factors, including individual characteristics (such as age, gender, abnormal liver function, abnormal kidney function, pregnancy status, and stress state) and other factors (administration methods, drug combination, combined physical therapy, and exercise).^[63–74]^ These influencing factors also include the impact of acupuncture on drug pharmacokinetics,^[75,76]^ specifically divided into acupoints and non-acupoints. Acupuncture has different effects on pharmacokinetics.^[77–79]^ Acupuncture at different acupoints has different effects on pharmacokinetics.^[80,81]^ Different operational variables in acupuncture, such as manual acupuncture, electroacupuncture frequency, and waveform, or laser acupuncture, also have different effects on pharmacokinetics.^[82–86]^ Compared to the abstract and difficult-to-verify meridian-acupoint system, pharmacokinetics offers a more concrete and direct framework for research. Notably, the same acupoint can influence the pharmacokinetics of different drugs in distinct ways. This review focuses on summarizing the effects and mechanisms of acupuncture at ST36 on the pharmacokinetics of different drugs, aiming to raise awareness about the interactions between acupuncture and drugs and their impact on the human body. While there is in-depth research on drug-drug and herbal-drug interactions,^[87–94]^ studies exploring the effects of acupuncture on pharmacokinetics remain limited and lack systematic analysis. However, in clinical practice, the simultaneous use of acupuncture and medicine occurs widely.

In this review, we collected and analyzed the effects of acupuncture or electroacupuncture at ST36 on pharmacokinetics (absorption, distribution, metabolism, and excretion). We aim to elucidate the intrinsic mechanisms of these effects, enhance understanding of acupuncture therapy and ST36, and provide warnings about the adverse effects of its clinical application. Acupuncture is widely used in clinical practice and is increasingly accepted by individuals with illnesses and healthy people.^[95–97]^ Many patients use acupuncture concurrently with medications, and some clinicians prefer prescribing Chinese medicine alongside acupuncture. Consequently, the interaction between acupuncture and medicine warrants close attention. The interaction between acupuncture and medicine may cause potential damage to the human body. This article emphasizes the interaction between acupuncture and medicine and their effects on the human body.

### 3.1. Effect of acupuncture at ST36 on drug absorption

Drug absorption is the process by which a drug enters the circulation from its site of administration. For oral drugs, the primary site of absorption is the gastrointestinal tract. Many factors influence drug absorption, including sex, age, race, disease, and gastrointestinal conditions.^[98–100]^ The absorption process depends on the drug’s permeability and dissolution. Acupuncture does not directly change a drug’s physicochemical properties; rather, any impact on absorption is mediated through physiological changes (e.g., gastrointestinal motility).^[101,102]^ Acupuncture at ST36 is well-known to influence gastrointestinal function. Research by Lee et al found that acupuncture at ST36 can activate brain centers and increase vagus nerve efferent activity, thereby improving gastric motility and secretion.^[22]^ Wu et al reported that acupuncture at ST36 increased the absorption of the main components of Schisandra chinensis in rats, potentially due to stimulated gastrointestinal motility and improved digestive function (Schisandra chinensis contains active lignans such as schisandrin A and B, which were measured in that study).^[50]^ Studies by Ming et al found that electroacupuncture at ST36 significantly accelerated the absorption of acetaminophen (APAP) in rats – with treated rats reaching a higher peak plasma concentration of APAP faster than controls – which could potentially lead to excessive drug absorption and associated adverse effects.^[103,104]^ Follow-up dose-controlled studies revealed that at high doses of APAP, electroacupuncture at ST36 led to a significantly greater absorption and bioavailability of the drug, accompanied by a reduction in glutathione (GSH) and related detoxifying enzymes in the liver.^[105]^ GSH is crucial for antioxidant defense and detoxification, and its depletion is associated with liver injury. Indeed, rats receiving high-dose APAP with ST36 acupuncture showed signs of liver damage, likely resulting from the increased APAP absorption and subsequent GSH depletion. These findings suggest that while acupuncture at ST36 can enhance the absorption of certain drugs, it may also increase the risk of toxicity for drugs with narrow safety margins when given at high doses.^[106–109]^ *Tripterygium wilfordii* is a common Chinese herbal medicine whose main component, triptolide, is widely used in clinical practice because of its excellent anti-inflammatory, antitumor, and immune-modulating functions. However, due to its significant toxicity (reproductive, hepatic, renal, and myocardial toxicity), its safety, nontoxic dose range, exposure routes, and metabolic pathways have all attracted attention.^[110–114]^ Bin et al found that acupuncture at ST36 can significantly increase the absorption of triptolide in rats; however, the mechanism has not been elucidated.^[115]^ Further studies by Hao and Bin showed that acupuncture could significantly increase the blood concentration of oral triptolide, which may change its nontoxic dose range due to the promotion of gastrointestinal absorption of triptolide through acupoint stimulation.^[116,117]^ However, the mechanism of this effect is complex. Changes in medication status caused by acupuncture may alter drug metabolism parameters, so our medication regimens must also be adjusted, especially for drugs such as triptolide that are inherently toxic. Ignoring the interaction between needles and drugs may lead to differences in clinical benefits. This phenomenon highlights the need for cautious use and systematic research. Additionally, acupuncture at ST36 can also affect the absorption of drugs by local tissues and organs. Senna-Fernandes et al found that electroacupuncture at ST36 enhanced the absorption of Na99mTcO4 by the thyroid gland, with the most pronounced effect observed at a frequency of 100 Hz, which may be related to acupuncture at ST36 affecting the neuroendocrine system.^[82]^

### 3.2. Effect of acupuncture at ST36 on drug distribution

Drug distribution refers to the process by which drugs circulate in the bloodstream after absorption and then distribution to the interstitial fluid and intracellular fluid. The pharmacological activity of a drug primarily depends on its distribution to its target site. Drug distribution directly affects the effect of the drug on tissues, organs and the body. Since measuring drug concentration in tissues and organs is challenging, circulating plasma drug levels are often used to estimate the degree of drug exposure. However, plasma drug concentrations do not always correspond to distribution in tissues and organs and may not directly reflect the distribution of the drug.^[118]^ Drugs with targeted distributions may produce toxic side effects if the concentration is too high in the target organ, while insufficient concentrations could undermine therapeutic efficacy. Therefore, it is particularly important to pay attention to drug distribution. Previous studies have confirmed that acupuncture affects drug distribution, which we have compiled and analyzed. Frederico et al^[83]^ found that laser stimulation of ST36 significantly increased the concentration of Na99mTcO4 in the thyroid gland without affecting its levels in other organs and tissues. These effects were considered to be due to the regulation of acupuncture on visceral reflexes, which influence the central and autonomic nervous systems.^[82,83,115,119]^ It is hypothesized that stimulation of ST36 affects the distribution of Na99mTcO4, enhancing its exposure concentration in the thyroid gland or altering thyroid function, promoting greater uptake of Na99mTcO4. However, these hypotheses lack in-depth research, highlighting the need for further investigation.

Lu et al and Ji et al reported that acupuncture at ST36 significantly altered the tissue distribution of Schisandra chinensis lignans. Normally, the highest concentrations of these lignans (e.g., schisandrin, deoxyschisandrin, schisandrin B) occur in the liver. With ST36 acupuncture, however, the relative distribution shifted: concentrations in lung tissue increased markedly, while liver concentrations were relatively lower.^[50,120]^ Schisandra chinensis is known for its antioxidant and anti-fatigue effects; this altered distribution could potentially enhance the herb’s activity in the lungs (beneficial for its traditional antitussive use). Notably, no toxic effects were observed from this redistribution, as Schisandra lignans are not highly toxic. If a drug with higher toxicity were to exhibit similar prolonged tissue retention, there could be a risk of accumulation and adverse effects. Hao et al reported increased triptolide exposure with ST36 stimulation, indicating the need for caution with toxic drugs. In summary, acupuncture at ST36 may influence drug distribution by modulating central and autonomic nervous system outputs, which in turn can alter regional blood flow or the expression of membrane transporters in certain organs. Ren et al even demonstrated with an acupoint-targeted hydrogel that stimulation at ST36 could synergistically improve the targeted delivery of triptolide, reducing its hepatotoxicity in a rat arthritis model. It should be noted, however, that this particular result was achieved via an acupoint injection system and may not directly translate to conventional acupuncture with oral drugs.^[121]^ Nevertheless, these findings collectively highlight that significant changes in drug distribution can occur with ST36 stimulation. Clinicians should monitor for possible distribution-related effects when acupuncture is combined with potent medications.

For instance, studies by Wang et al and Chen et al showed that acupuncture at ST36 significantly increased the blood concentration and exposure average plasma concentration-time curve of the inherently toxic compound triptolide (from *T wilfordii*) after oral administration. This finding suggests a higher risk of drug toxicity due to enhanced gastrointestinal absorption of triptolide under acupuncture. Hao et al reported increased triptolide exposure with ST36 stimulation, indicating the need for caution with toxic drugs.^[116,122]^ In summary, acupuncture at ST36 may influence drug distribution by modulating central and autonomic nervous system outputs, which in turn can alter regional blood flow or the expression of membrane transporters in certain organs. Ren et al even demonstrated with an acupoint-targeted hydrogel that stimulation at ST36 could synergistically improve the targeted delivery of triptolide, reducing its hepatotoxicity in a rat arthritis model. It should be noted, however, that this particular result was achieved via an acupoint injection system and may not directly translate to conventional acupuncture with oral drugs. Nevertheless, these findings collectively highlight that significant changes in drug distribution can occur with ST36 stimulation. Clinicians should monitor for possible distribution-related effects when acupuncture is combined with potent medications.

### 3.3. Effect of acupuncture at ST36 on drug metabolism and clearance

Drug clearance encompasses both metabolism and excretion processes that remove a drug from the body. Metabolism typically occurs in the liver (as well as in plasma, kidneys, etc), transforming drugs into more water-soluble metabolites, often inactivating them. Excretion routes include the kidneys (primary route for most drugs), bile, lungs, and sweat. Changes in metabolism or excretion directly affect drug levels and effects, and many factors (genetic, physiological, environmental) can modulate these processes.

Acupuncture at ST36 has been investigated for its influence on drug-metabolizing enzymes and clearance rates. For example, Feng et al found that combining ST36 acupuncture with curcumin therapy improved outcomes in a liver fibrosis model, suggesting a potential role in modulating drug disposition in diseased liver.^[123]^ In contrast, Wu et al observed that ST36 acupuncture (manual or electroacupuncture) did not significantly affect the metabolic rate of aspirin or the formation of its metabolites.^[86]^ These results indicate that the impact of ST36 on drug metabolism may vary depending on the drug and context. In general, the effects of acupuncture at ST36 alone differ from those when acupuncture is combined with a specific drug; each drug–acupuncture combination can yield different outcomes. This underscores the need to examine each case individually.

Studies focusing on APAP provide more insight. Meng et al reported that electroacupuncture at ST36 significantly slowed the metabolism of APAP, resulting in a higher plasma average plasma concentration-time curve and prolonged half-life compared to APAP alone. The increase in APAP exposure with acupuncture was more pronounced at higher doses.^[109]^ Biochemically, rats receiving both high-dose APAP and ST36 stimulation showed elevated serum glutathione S-transferase activity and reduced hepatic GSH levels, indicating that acupuncture might affect phase II detoxification pathways (glutathione S-transferase is involved in APAP conjugation). These results suggest that ST36 acupuncture can impair the clearance of APAP, potentially leading to drug accumulation and increased risk of hepatotoxicity at high doses. In our study, rats in the combined APAP + ST36 group indeed exhibited more severe liver injury markers than those given APAP alone. The likely mechanism is that acupuncture altered hepatic enzyme activity or blood flow, thus reducing APAP clearance and allowing toxic metabolites to accumulate. (Of note, most previous studies indicate acupuncture at ST36 tends to reduce oxidative stress and generally does not deplete GSH. The APAP findings reveal an exception where acupuncture’s effect in the presence of an overdose unmasked a potential safety concern).

Another aspect of clearance is renal excretion. Acupuncture could, in theory, influence renal function (e.g., via sympathetic or endocrine pathways) and thereby modify drug excretion. For instance, acupoint stimulation might affect glomerular filtration or tubular reabsorption. If acupuncture were to reduce renal clearance of a drug, it would cause that drug to remain longer in the system. However, such effects remain speculative, and any influence of acupuncture on renal drug excretion would apply mainly to drugs that are primarily cleared by the kidneys. To date, there is limited direct evidence of acupuncture significantly altering renal excretory function for drugs. One study on anesthetized rats suggested that electroacupuncture could attenuate renal oxidative stress, but it did not measure drug excretion. Overall, a decrease in drug clearance (whether metabolic or renal) will cause drug accumulation and changes in the pharmacokinetic profile, which may necessitate dose adjustments if acupuncture is used concurrently. In clinical practice, until more data are available, it would be prudent to monitor patients’ drug levels or effects when combining acupuncture with medications that have a narrow therapeutic index or rely on precise dosing. We have summarized the documented pharmacokinetic interactions between ST36 acupuncture and various drugs in Table 1.

**Table 1:** Effect of ST36 acupuncture on the pharmacokinetics of different drugs (with key findings and proposed mechanisms).^[50,82–84,86,103,104,115,116,119,120,122]^

Drug (reference)	PK changes with ST36 acupuncture	Proposed mechanism
Aspirin^[86]^	No significant change in metabolism or pharmacokinetic parameters (no notable difference in AUC, *C*_max_, or T_1/2_)	None observed (acupuncture did not alter aspirin’s metabolic pathways)
Triptolide^[116,122]^	Increased absorption and plasma exposure – acupuncture at ST36 significantly ↑ *C*_max_ and AUC of triptolide (oral) in rats, indicating higher systemic exposure. *Potential toxicity risk*: elevated drug levels correlated with greater hepatic toxicity markers	Enhanced gastrointestinal motility and absorption (vagus nerve stimulation) leading to greater drug uptake; possible inhibition of first-pass metabolism
Technetium-99m Pertechnetate (Na^99m^TcO_4)^[82,83,115,119]^	Altered distribution-ST36 acupuncture caused ↑ uptake of Na*^99m^*TcO_4 in thyroid tissue (e.g., thyroid radioactivity counts significantly increased ~20–30% vs control), with no change in other organs. Overall bioavailability may increase for thyroid-targeting substances	Visceral reflex via autonomic nervous system targeting the thyroid; increased regional blood flow or membrane transporter activity in thyroid gland
Schisandra chinensis *lignans*^[50,120]^	Increased absorption and altered distribution – acupuncture ↑ plasma AUC and *C*_max_ of schisandrin and related lignans; T significantly ↓ (faster absorption). Organ distribution shifted: higher concentrations in lungs, relatively lower in liver. No acute toxicity observed	Improved gastrointestinal absorption (enhanced peristalsis and secretion), and neural regulation of tissue uptake (possibly via altered expression of drug transporters or blood flow distribution to lung tissue)
Acetaminophen (APAP)^[103,104]^	Faster absorption but slower clearance at high dose-ST36 electroacupuncture shortened APAP T and ↑ initial absorption rate (higher early concentrations). In high-dose scenarios, acupuncture significantly ↑ AUC (e.g., AUC increased by ~30–40% in 1 study’s acupuncture group vs control) and prolonged APAP elimination. Associated with ↓ GSH levels and ↑ liver enzyme release (greater liver injury) in rats	Vagal stimulation improving gastric emptying (faster absorption); reduced metabolic clearance due to modulation of liver enzymes (e.g., phase II conjugation via GST) and hepatic blood flow. GSH depletion suggests acupuncture + APAP may overwhelm antioxidant defenses, slowing detoxification
Isoflurane Anesthetic (minimum alveolar conc., MAC)^[84]^	Reduced anesthetic requirement – Electroacupuncture at ST36 decreased the MAC of isoflurane in dogs by ~10–15% (meaning less isoflurane was needed to achieve the same level of anesthesia) according to the cited study	Acupuncture’s analgesic and sedative effects likely enhanced the anesthetic potency, possibly through endorphin release or autonomic modulation, thereby reducing the amount of anesthetic needed. (This is a pharmacodynamic interaction rather than a pharmacokinetic change)

↑ = increase; ↓ = decrease. Values are described qualitatively based on significance reported; exact percentages are given where available from studies.

## 4. Conclusions

Traditional Chinese medicine is an ancient practice rooted in Chinese philosophy, influenced by both natural sciences and humanities. Integrating traditional Chinese medicine (including acupuncture) with modern scientific research is important to uncover its mechanisms and to enhance its evidence-based application. Acupuncture, as a widely used therapy, offers substantial health benefits but, like any treatment, requires consideration of its indications and safety. While classical acupuncture theory outlines specific indications and some contraindications, there is a lack of guidelines regarding precautions when acupuncture is combined with drug therapy. Research on acupuncture’s mechanisms and effects should therefore address not only efficacy but also safety in the context of concomitant medication use. An objective understanding of acupuncture’s benefits and limitations will pave the way for a robust safety evaluation system in future research.

The combination of acupuncture and conventional medicine has broad prospects. The interactions between acupuncture and medications deserve careful study to maximize therapeutic effects while ensuring safety. Developing a theoretical and practical framework for acupuncture–drug co-therapy is essential. Based on our review, we recommend that clinicians take a thorough medication history before administering acupuncture. If a patient is on a potentially toxic medication (with a narrow therapeutic window), it may be prudent to avoid performing acupuncture simultaneously with drug administration or to schedule acupuncture and medication at a safe interval apart. When both treatments are necessary, clinicians could consider monitoring the patient’s drug levels or clinical signs closely. If feasible, measuring blood drug concentrations in patients receiving both acupuncture and medication could provide valuable data for future research and clinical guidance.

This review focused on pharmacokinetic changes associated with acupuncture at ST36. Many other commonly used acupoints may likewise affect drug disposition and merit investigation. We acknowledge that our review includes a number of studies from Chinese literature; this regional focus may limit generalizability. Nonetheless, the issues highlighted should draw the attention of healthcare providers worldwide who use acupuncture alongside pharmacotherapy. We call on international researchers to further explore acupuncture–drug interactions through well-designed basic and clinical studies. Accumulating more evidence (including case reports of adverse or beneficial interactions) will help in creating a comprehensive database of acupoint–pharmacokinetic interactions. Ultimately, this knowledge will inform clinical practice, improve the safety of integrative medicine, and prevent harm to patients due to unanticipated acupuncture–drug interactions.

## Acknowledgments

We thank all the researchers whose work contributed to this review.

## Author contributions

**Conceptualization:** Xinyu Liu, Wenfeng Zhang, Yonggang Wang.

**Data curation:** Xinyu Liu.

**Formal analysis:** Cheng Li.

**Investigation:** Cheng Li.

**Methodology:** Yinghua Hu.

**Project administration:** Wenfeng Zhang.

**Supervision:** Yinghua Hu, Cheng Li, Yonggang Wang.

**Writing** – **original draft:** Xinyu Liu, Yifan Zhao.

**Writing** – **review & editing:** Xinyu Liu, Yifan Zhao.
